# Miniaturized tri-notched wideband bandpass filter with ultrawide upper stopband suppression

**DOI:** 10.1038/s41598-021-92394-7

**Published:** 2021-06-21

**Authors:** Chen-Hao Wang, Xiao-Min Shi

**Affiliations:** 1 Department of Electronic Engineering, Xi’an University of Technology, Xi’an, 710048 People’s Republic of China; 2 Department of Communication Engineering, Xi’an Shiyou University, Xi’an, 710065 People’s Republic of China

**Keywords:** Engineering, Physics

## Abstract

Stepped impedance resonator (SIR) and its derivative resonators are widely used in the design of microwave filters. However, many spurious modes will be introduced into the stopband, resulting in lower upper stopband suppression and performance degradation. Based on the principle of slit line, a method to enhance the upper stopband suppression is proposed and verified by a miniaturized tri-notched wideband bandpass filter based on stub loaded ring resonator (SLRR) and shorted-stub loaded SIR (SSLSIR). The wideband is formed by coupling SLRR and interdigital lines, which has a rectangular DGS on the back of the substrate. Three notched bands with controllable positions in the passband can be produced by inserting two different SSLSIRs inside and outside the SLRR. Four slit lines are loaded on the low impedance stub of SSLSIR to adjust the high-order modes close to the transmission zeros (TZs). The operating frequency of the filter is 2.2–7.6 GHz, and the three notched bands are located at 2.97 GHz, 5.75 GHz and 6.46 GHz, respectively. The measurement results show that the − 20 dB enhanced upper stopband of the filter can reach 32 GHz, which proves that the filter has the characteristic of ultrawide upper stopband suppression while keeping the miniaturization.

## Introduction

Wideband communication system is widely used in information industry, such as indoor positioning system, wideband radar and so on. The working frequency of other transmitters will inevitably fall into the passband of the wideband receiver, which will affect the demodulation ability of the system, such as the signal amplitude after pulse compression in radar system. Therefore, wideband filters with notched bands are still in great need. Many methods have been reported to realize one or more notched frequencies in the passband, such as ${\lambda \mathord{\left/ {\vphantom {\lambda 2}} \right. \kern-\nulldelimiterspace} 2}$ resonators^1^, exponential tapered impedance line^2^, embedded open stubs^3–5^, varactor-loaded shorted-slots^6^, asymmetric structure^7^, terminated capacitance^8^, signal interaction between main and minor paths^9,10^, folded coupling line^11,12^, and multi-mode resonator (MMR)^13–18^. Most of them use transmission poles and zeros to achieve good wideband performance and controllable notched bands, which can effectively suppress interference^19–26^. However, MMR can not only generate transmission poles that constitute wideband, but also limit the suppression range of the upper limit of stopband. Two transmission zeros are introduced into the stopband by the tapped lines loaded on the input and output structures to suppress the spurious modes^9^. The results show that the upper limit of stopband can be increased to 8.2 times of ${\text{f}}_{0}$. However, the use of stopband expansion technology will affect the performance of passband. A dual-mode fractal bandstop filter cascaded low-pass filter is designed to increase the stopband range without affecting the wideband characteristics, but inevitably expands the area of the whole circuit^20,21^.

In addition to the implementation of wideband filters with multiple notched bands, it is also important to achieve a higher upper stopband suppression range, which is difficult to achieve at the same time.

In this paper, a miniaturized tri-notched wideband bandpass filter based on SLRR and SSLSIR with the characteristic of ultrawide upper stopband suppression is proposed. The interdigital line is coupled with the open stub at both ends of SLRR, and DGS is set below the coupling gap to form a wide passband response. Two type SSLSIRs are placed inside and outside the ring of SLRR and three controlled notched bands are introduced into the passband. Slit lines are vertically loaded on the low impedance section of SSLSIR to adjust the high-order mode to meet the four TZs outside the passband. The -20 dB enhanced upper stopband of the filter can reach 32 GHz. The novelty of this paper is that by studying the principle of slit line, the characteristics of miniaturization, ultrawide upper stopband, wide passband and independently adjustable multi-notched bands can be obtained at the same time to achieve a multi-functional high-performance microwave filter.

## Results

**Figure 1:**
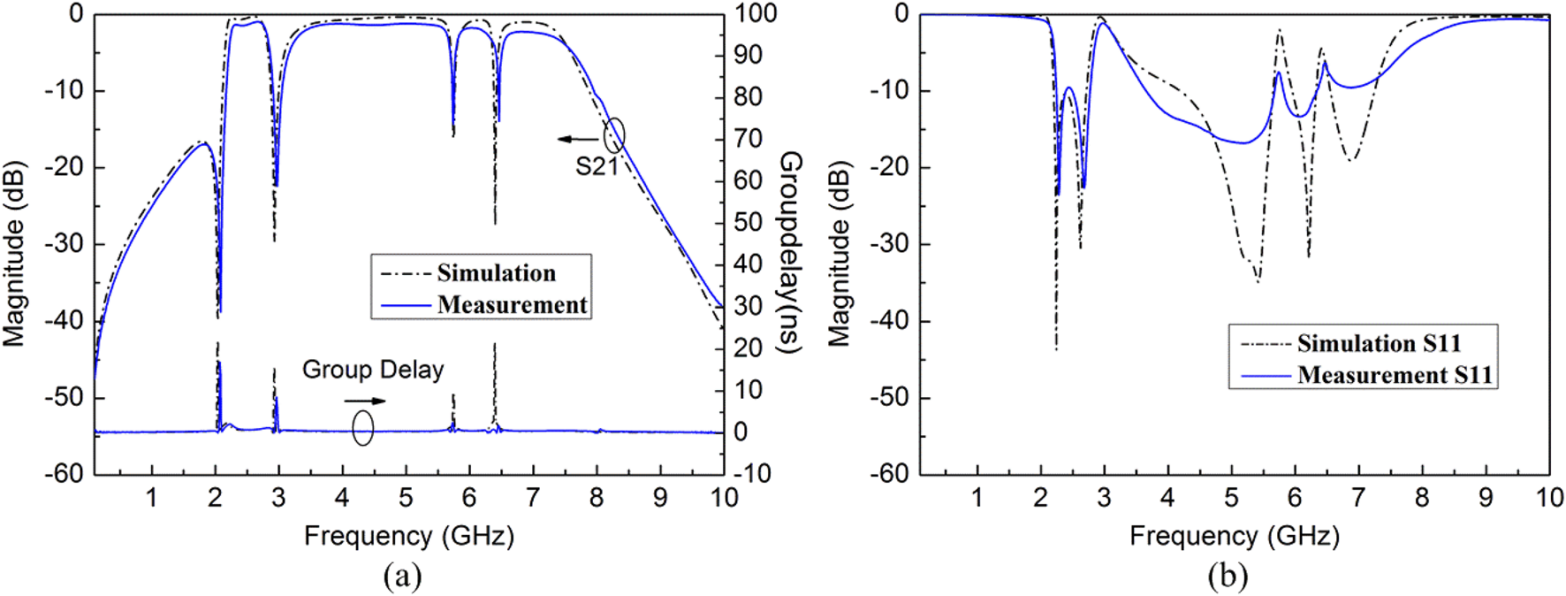
The simulation and measurement results of the proposed filter, (**a**) S21 and group delay (**b**) S11.

The demonstrated wideband bandpass filter with tri-notched band is simulated and optimized on Rogers 4350B with thickness of 0.5 mm and dielectric constant of 3.48. The final optimized dimensions of the proposed filter in millimeters can be summarized as follows: L1 = 13.1, W1 = 1.5, L2 = 3, W2 = 2.4, L3 = 3.85, W3 = 3.0, L4 = 8.0, W4 = 0.5, L5 = 10.5, W5 = 2.0, L6 = 11.9, W6 = 1.0, L7 = 5.3, W7 = 1.0, L8 = 1.3, W8 = 1.3, L9 = 1.3, W9 = 2.5, g1 = 0.4, g2 = 0.3, g3 = g4 = 0.5, S1 = 0.2, S2 = 0.2, d1 = 1.4, d2 = 1.7. The size of the four vias on SSLSIRs is 0.5 mm. The simulation and measurement results of S parameters are shown in Fig. 1.

The center frequency of the filter is 4.9 GHz, and the − 3dB bandwidth is from 2.2 to 7.6 GHz. The maximum insertion loss is − 2.3 dB and the reflection coefficient is better than − 12 dB in wideband, except for the three notched bands with − 10 dB notched bandwidth ranging from 2.85 to 3.05 GHz, 5.72 GHz to 5.83 GHz and 6.42 to 6.51 GHz. The maximum insertion loss of the three notched bands is − 22.3 dB at 2.97 GHz, − 14.3 dB at 5.74 GHz and − 13.9 dB at 6.46 GHz, which indicates that the tri-notched wideband filter has good performance in suppressing passband interference. Due to the introduction of transmission zeros at 2.08 GHz and 10.2 GHz, sharp skirt is achieved to improve selectivity. The group delay is between 0.7 ns and 5 ns, which indicates that the passband linearity is good. The small deviation between simulation and measurement may be caused by the inevitable difference between filter sample and the simulation model, including tiny manufacturing error, dielectric constant shift, welding error and the parasitic effect of coaxial cable and connector.

**Figure 2:**
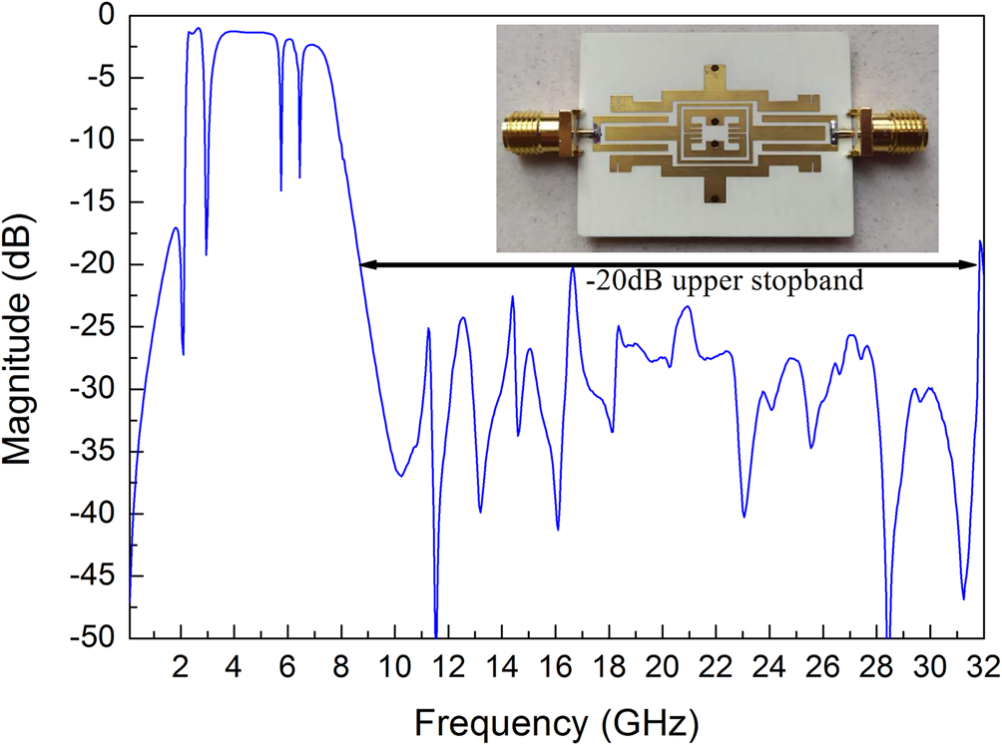
The proposed tri-notched wideband filter with ultrawide upper stopband up to 32 GHz.

Figure 2 shows the S21 curve of the proposed filter with an ultrawide upper stopband range. Its obvious advantage is that the spurious frequencies are disturbed to the positions of TZs due to the influence of slit lines loaded on the SSLSIRs. The proposed filter achieves an ultrawide upper stopband with − 20 dB suppression up to 32 GHz while maintaining a good wideband and tri-notched characteristic. The results show that the proposed filter has novel performance, which can realize the functions of ultrawide stopband suppression, adjustable multi-notched bands and wideband characteristic, while maintaining a compact size.

## Discussion

**Figure 3:**
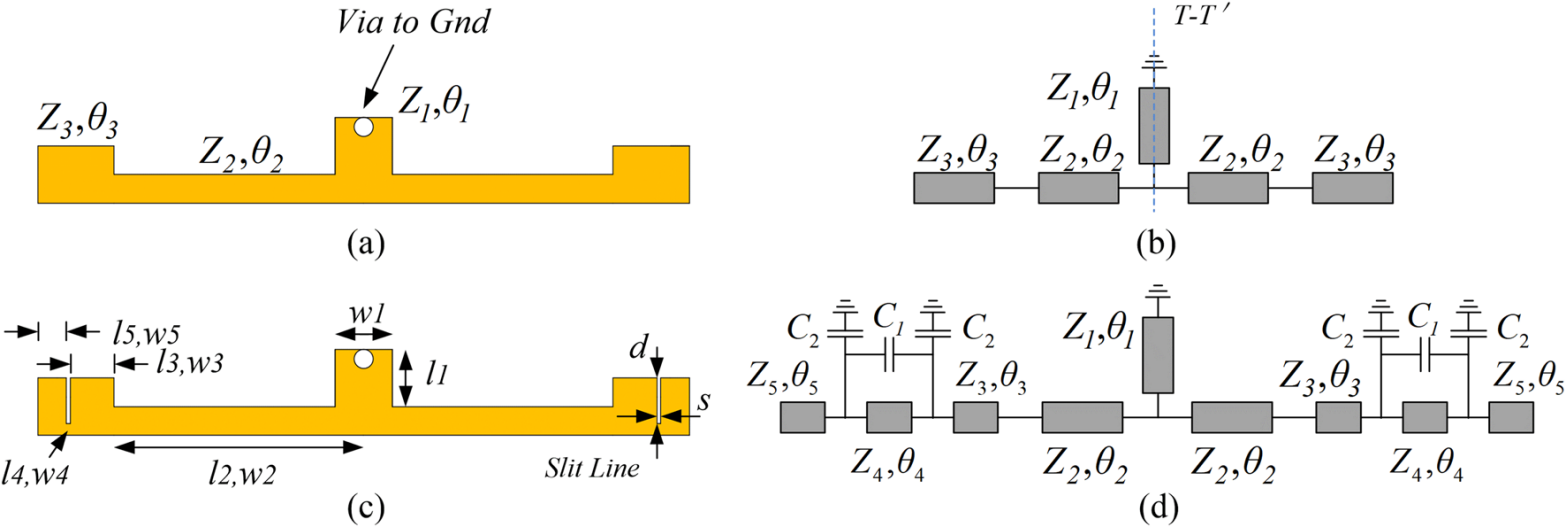
(**a**) The geometry of SSLSIR without slit line. (**b**) The equivalent circuit of SSLSIR without slit line. (**c**) The geometry of SSLSIR with two slit line loaded. (**d**) The equivalent circuit of SSLSIR with two slit line loaded.

**Figure 4:**
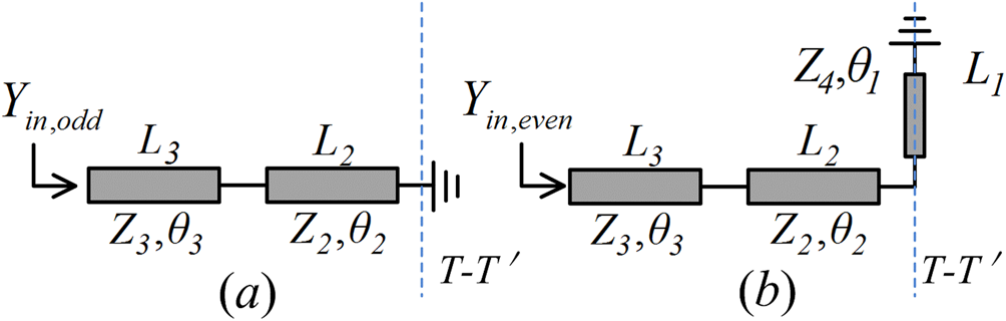
The equivalent odd–even circuit of SSLSIR without slit line loading, (**a**) odd mode and (**b**) even mode.

**Figure 5:**
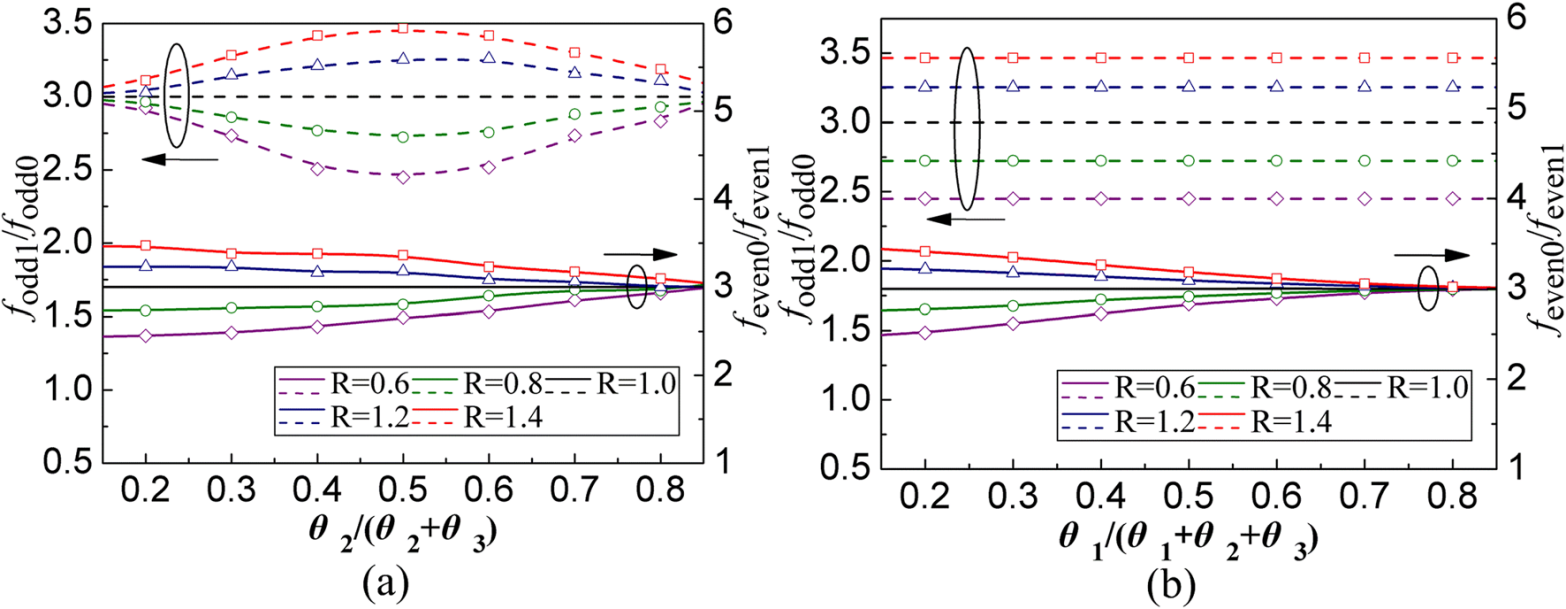
The first odd-mode fodd1 normalized by fodd0 and the first even-mode feven1 normalized by feven0 against various of (**a**) $\theta _{2} /(\theta _{2} + \theta _{3} )$ and (**b**) $\theta _{1} /(\theta _{1} + \theta _{2} + \theta _{3} )$.

The geometry and equivalent circuit of SSLSIR without slit lines loading are depicted in Fig. 3a and b. It is symmetric to the plane T–T' and the odd-even mode method can be used to analyze the fundamental and spurious modes. The substrate used in this section is Rogers4350B with a thickness of 0.5 mm and a dielectric constant of 3.48. The simulated data is achieved by HFSS 15.0. The equivalent odd-even circuit of SSLSIR without slit line loading is shown in Fig. 4. In order to simplify the analysis process, it is assumed that the odd and even resonance conditions can be derived from the short circuit and open circuit plane T–T', which are expressed as Eqs. (1) and (2):

1 $${{1/R = tan\theta }}_{{{2}}} {{tan\theta }}_{{{3}}}$$

2 $${{1/R = tan(\theta }}_{{{1}}} + {{\theta }}_{{{2}}} {{)tan\theta }}_{{{3}}}$$

where $R = Z_{2} /Z_{3}$ is the impedance ratio of the high impedance section to the low impedance section. The position of the odd–even spurious mode can be adjusted by controlling the impedance ratio R or the length ratio $\theta _{{{2}}}$ to $\theta _{{{2}}} + \theta _{{{3}}}$, $\theta _{1}$ to $\theta _{1} + \theta _{{{2}}} + \theta _{{{3}}}$ with a fixed $\theta _{{{1}}} = \theta _{3}$, as shown in Fig. 5. It can be seen that in order to obtain a wider upper stopband in the filter design, it is necessary to keep the higher-order modes away from the foundational modes, and a larger R value should be selected.

The geometry and equivalent circuit of SSLSIR loaded with two slit lines are illustrated in Fig. 3c and d. The obvious difference between Fig. 3d and b is that the slit line divides the low impedance part of SSLSIR into three parts, ${Z}_{3} ,\theta _{3}$,${{{Z}}}_{4} ,{{\theta }}_{4}$ and ${{{Z}}}_{5} ,{{\theta }}_{5}$, respectively. Slit line introduces one coupling capacitance C1 and two bypass capacitors C2 in the equivalent circuit that connected in series and parallel with section ${{\theta }}_{4}$. The resonant modes can also be analyzed by inserting electric wall and magnetic wall respectively at the symmetric interface T–T' under the condition of weak coupling. According to the theory of microwave network, the input admittance Y of the odd–even mode circuits can be derived from $[ A ]$ matrix which expressed as Eq. (3):

3 $$\begin{aligned} Y_{{odd}} & = \frac{{A_{{21\_odd}} Z_{l} + A_{{22\_odd}} }}{{A_{{11\_odd}} Z_{l} + A_{{12\_odd}} }} \\ Y_{{even}} & = \frac{{A_{{21\_even}} Z_{l} + A_{{22\_even}} }}{{A_{{11\_even}} Z_{l} + A_{{12\_even}} }} \\ \end{aligned}$$

where $Z_{l}$ is the load condition of odd–even equivalent circuit of SSLSIR and $A_{{11}}$, $A_{{21}}$, $A_{{12}}$, $A_{{22}}$ are the elements of $[ A ]$ matrix of a fully cascaded odd–even circuit respectively. After derivation, the input admittances Y of odd–even modes are obtained as formula (4) and (5). The capacitance C1 and C2 can be calculated approximately according to the method in references^27–30^.

**Figure 6:**
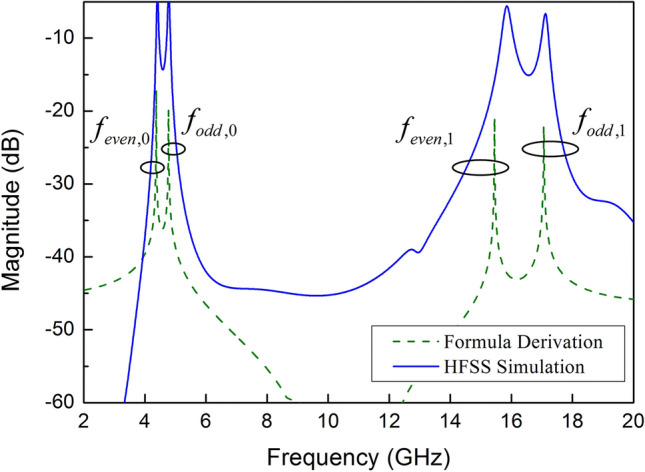
The resonance mode curves derived from the formula and HFSS simulation of SSLSIR loaded with slit line.

It is well known that the resonant modes can be obtained by making the input admittance of odd–even equivalent circuit equals to 0. In order to prove the correctness of the derived formula, when l1 = 1.3 mm, l2 = 4.3 mm, l3 = 1.8 mm, l4 = 0.2 mm, l5 = 1 mm, w1 = 1.3 mm, w2 = 0.8 mm, w3 = 2 mm, w4 = 0.6 mm, w5 = 2 mm, d = 1.4 mm, the HFSS simulation curve is compared with the formula derived curve, as shown in Fig. 6. It can be found that the fundamental frequencies and the first-order resonant modes almost coincide with each other at 4.4 GHz, 4.8 GHz, 15.9 GHz, 17.1 GHz, except tiny discrepancy appears at $f_{{even,1}}$ which is caused by some factors that are not taken into account during modeling. The two curves are in good agreement, which verifies the validity of the equivalent model and Eqs. (4) and (5) in predicting the resonant modes of SSLSIR loaded with slit lines.

4 $$Y_{{odd}} = \frac{{A_{{22\_odd}} }}{{A_{{12\_odd}} }}$$

$$\begin{aligned} A_{{22\_odd}} & = - Z_{3} [(sin\theta _{5} + \omega Z_{3} C_{2} cos\theta _{5} )(tan\theta _{4} + \omega Z_{4} C_{1} + \omega Z_{4} C_{2} )(Rsin\theta _{2} cos\theta _{3} + sin\theta _{3} cos\theta _{2} ) \\ & \quad + [Z_{4} (sin\theta _{5} + \omega Z_{3} C_{2} \cos \theta _{5} ) + Z_{3} \cos \theta _{5} (tan\theta _{4} + \omega Z_{4} C_{1} )](\cos \theta _{2} \cos \theta _{3} - Rsin\theta _{3} sin\theta _{2} ) \\ & \quad - \omega Z_{3}^{2} C_{2} Z_{3} cos\theta _{5} (tan\theta _{4} + \omega Z_{4} C_{1} )(Rsin\theta _{2} cos\theta _{3} + sin\theta _{3} cos\theta _{2} ) \\ A_{{12\_odd}} & = jZ_{3} [Z_{3} \sin \theta _{5} (\tan \theta _{4} + \omega Z_{4} C_{1} ) - Z_{4} (\cos \theta _{5} - \omega Z_{3} C_{2} \sin \theta _{5} )](\cos \theta _{2} \cos \theta _{3} - R\sin \theta _{3} \sin \theta _{2} ) \\ & \quad + jZ_{3}^{2} (\cos \theta _{5} - \omega Z_{3} C_{2} \sin \theta _{5} )(\tan \theta _{4} + \omega Z_{4} C_{1} + \omega Z_{4} C_{2} )(R\sin \theta _{2} \cos \theta _{3} + \sin \theta _{3} \cos \theta _{2} ) \\ & \quad - j\omega Z_{3}^{3} C_{2} \sin \theta _{5} (\tan \theta _{4} + \omega Z_{4} C_{1} )(R\sin \theta _{2} \cos \theta _{3} + \sin \theta _{3} \cos \theta _{2} ) \\ \end{aligned}$$

5 $$Y_{{even}} = \frac{{A_{{22\_even}} }}{{A_{{12\_even}} }}$$

$$\begin{aligned} A_{{22\_even}} & = - Z_{3} [(\sin \theta _{5} + \omega Z_{3} C_{2} \cos \theta _{5} )(\tan \theta _{4} + \omega Z_{4} C_{1} + \omega Z_{4} C_{2} )[R\sin (\theta _{2} + \theta _{1} )\cos \theta _{3} + \sin \theta _{3} \cos (\theta _{2} + \theta _{1} )] \\ & \quad + [Z_{4} (\sin \theta _{5} + \omega Z_{3} C_{2} \cos \theta _{5} ) + Z_{3} \cos \theta _{5} (\tan \theta _{4} + \omega Z_{4} C_{1} )][\cos (\theta _{2} + \theta _{1} )\cos \theta _{3} - R\sin \theta _{3} \sin (\theta _{2} + \theta _{1} )] \\ & \quad - \omega Z_{3}^{2} C_{2} Z_{3} \cos \theta _{5} (\tan \theta _{4} + \omega Z_{4} C_{1} )[R\sin (\theta _{2} + \theta _{1} )\cos \theta _{3} + \sin \theta _{3} \cos (\theta _{2} + \theta _{1} )] \\ A_{{12\_even}} & = jZ_{3} [Z_{3} \sin \theta _{5} (\tan \theta _{4} + \omega Z_{4} C_{1} ) - Z_{4} (\cos \theta _{5} - \omega Z_{3} C_{2} \sin \theta _{5} )][\cos \theta _{2} \cos \theta _{3} - R\sin \theta _{3} \sin (\theta _{2} + \theta _{1} )] \\ & \quad + jZ_{3}^{2} (\cos \theta _{5} - \omega Z_{3} C_{2} \sin \theta _{5} )(\tan \theta _{4} + \omega Z_{4} C_{1} + \omega Z_{4} C_{2} )[R\sin (\theta _{2} + \theta _{1} )\cos \theta _{3} + \sin \theta _{3} \cos (\theta _{2} + \theta _{1} )] \\ & \quad - j\omega Z_{3}^{3} C_{2} \sin \theta _{5} (\tan \theta _{4} + \omega Z_{4} C_{1} )[R\sin (\theta _{2} + \theta _{1} )\cos \theta _{3} + \sin \theta _{3} \cos (\theta _{2} + \theta _{1} )] \\ \end{aligned}$$

**Figure 7:**
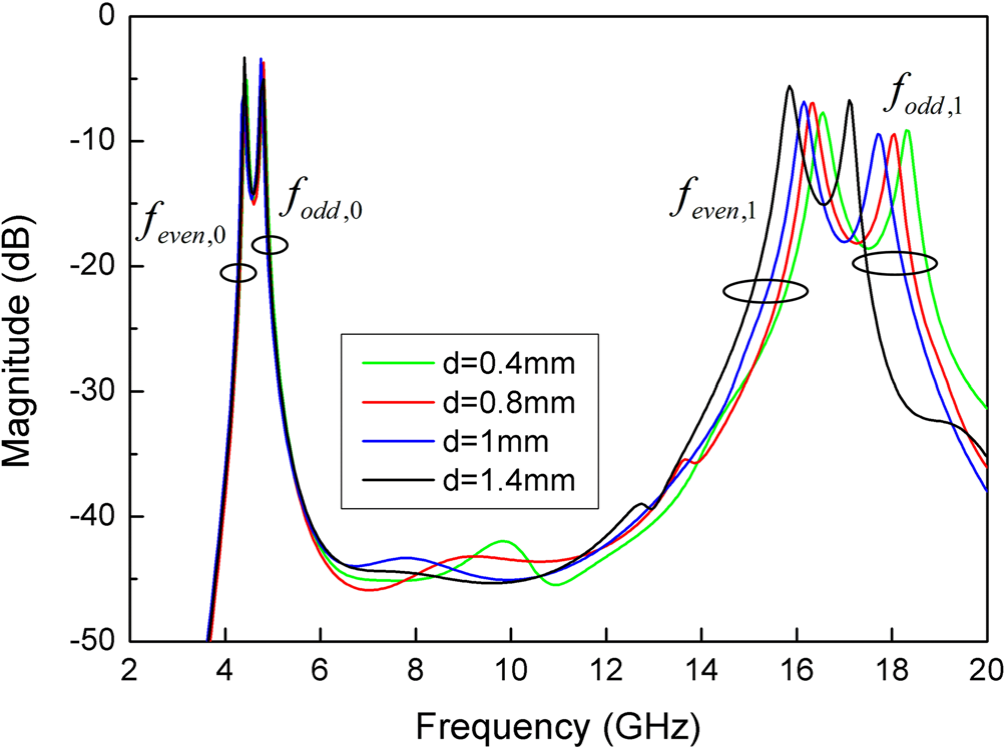
The simulated resonant modes curves of SSLSIR with different depth d of slit line.

**Figure 8:**
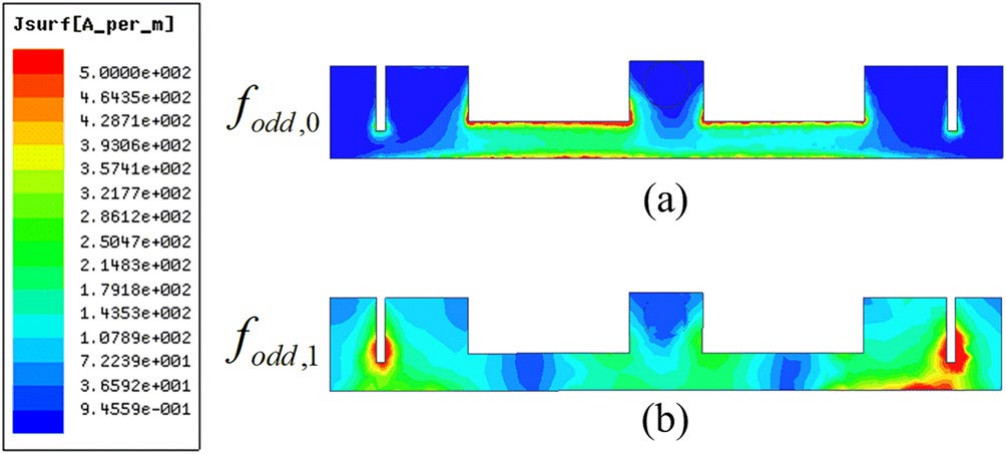
The surface current distribution of (**a**) fundamental odd mode and (**b**) 1st odd mode.

In addition, the dimension of slit line has a great influence on the properties of SSLSIR. The resonant modes curves of SSLSIR loaded with slit line are shown in Fig. 7. Except for one variable d, the dimension is the same as the previous section. The curves show that the 1st odd-even resonant mode is decreased from 18.3 to 17.1 GHz, 16.5 to 15.8 GHz when the depth d of slit line increases from 0.4 to 1.4 mm. Meanwhile the position of the fundamental modes remains unchanged. This characteristic is caused by the increase of Z4 and C1 in the equivalent circuit shown in Fig. 3d and can be illustrated by the surface current distribution of SSLSIR, as shown in Fig. 8. It is easy to point out that the slit line has no effect on the current distribution of fundamental mode but prolongs the current path of high-order spurious mode which is indicated by the red color region at the end of the slit line. The current path becomes longer. The wavelength corresponding to spurious mode increases, and the frequency of the spurious mode decreases accordingly. It shows the advantage of distributing spurious modes without affecting the fundamental mode. In other words, the controllable upper stopband and constant passband characteristics are composed of fundamental modes in the filter design.

**Figure 9:**
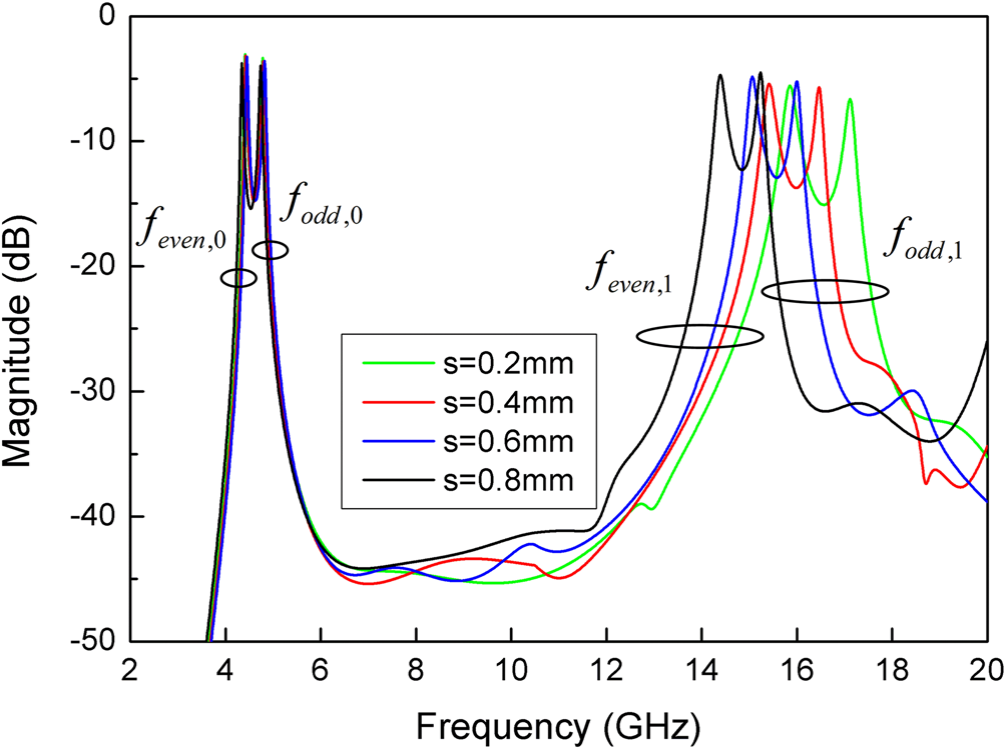
The simulated resonant modes of SSLSIR with different width s of slit line.

**Figure 10:**
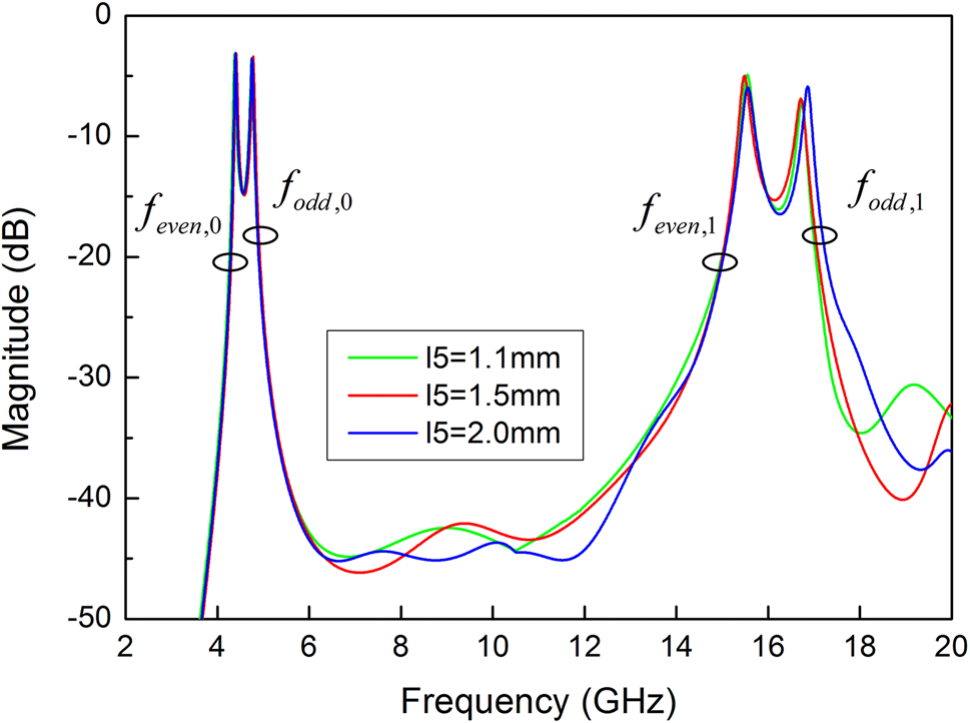
The simulated resonant modes of SSLSIR with different length l5 of slit line.

In order to further investigate the effect of slit line loaded on the resonator, Fig. 9 shows the resonant modes of SSLSIR with different width s of slit line. The 1st odd–even resonant modes are decreased from 17.1 to 15.2 GHz, 15.8 to 14.4 GHz with the width s increasing from 0.2 to 0.8 mm, while the foundational mode remains unchanged. The 1st odd–even mode deviation caused by width s is consistent with the effect of depth d. The spurious modes can be further decreased by controlling the width of the slit line after adjusting the depth of the slit line. For example, $f_{{odd,1}}$ deviates from 18.3 to 17.1 GHz and then from 15.8 to 14.4 GHz, according to the depth d increases from 0.4 to 1.4 mm and then the width s increases from 0.2 to 0.8 mm. Therefore, a wider range of spurious mode is achieved. Further, the resonant modes under different length l5 are depicted in Fig. 10. It can be found that the resonant mode almost keep invariant within a certain length of l5. This is because l5 does not determine the effective length of each mode current on the surface of the resonator, so the position of slit line at the open end of SSLSIR can be determined according to the optimization results.

## Methods

**Figure 11:**
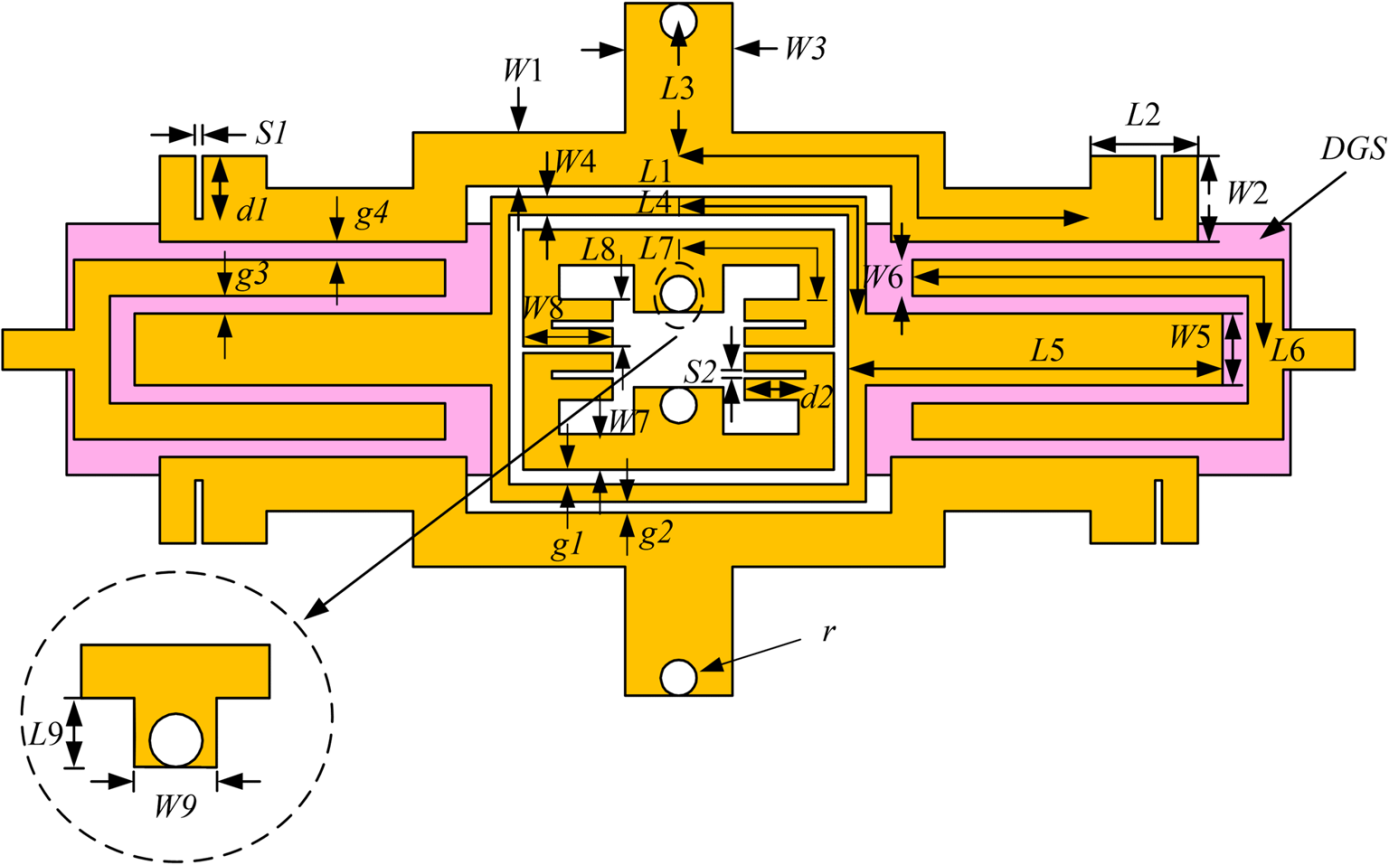
The layout of the proposed wideband BPF.

The layout of the proposed wideband filter is shown in Fig. 11. The interdigital lines are coupled with the open stubs at both ends of the SLRR with the set of DGS below the coupling gap to form a wide passband response. Two type SSLSIRs with the same shape but different dimensions are placed inside (SSLSIR type1) and outside (SSLSIR type2) the ring of SLRR to introduce three notched bands. Slit lines are loaded on the low impedance section of each SSLSIR in order to perturb spurious frequencies.

**Figure 12:**
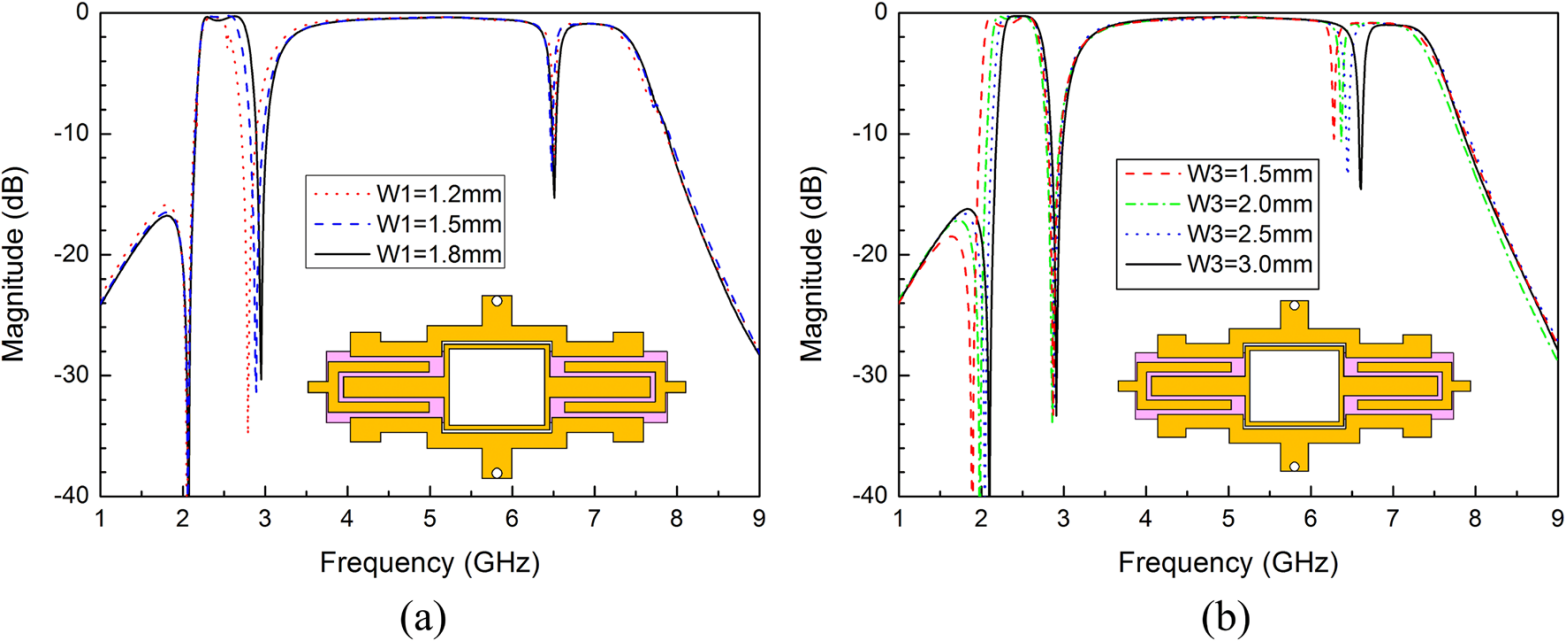
(**a**) The variable first notch frequency corresponding to different width W1. (**b**) The variable third notch frequency corresponding to different width W3.

The first and third notched bands are introduced by SSLSIR type2. The electrical length of the open stub L1 + L2 is set to a quarter wavelength to generate a transmission zero at the first notch frequency. Similarly, the third notched band is realized by the shorted stub L3, which has an electrical length of half wavelength and is combined with the parasitic inductance of the via and the mutual inductance between SLRR and SSLSIR type2. Since the positions of resonant modes are sensitive to the electrical length, the width W1 and W3 are used to fine tune the first and third notched bands, as shown in Fig. 12. The first notched band increases as stub width W1 increases with a fixed second notched band, and the third notched band has the same trend. Obviously, this characteristic leads to the independent adjustment between the first and third notched bands.

**Figure 13:**
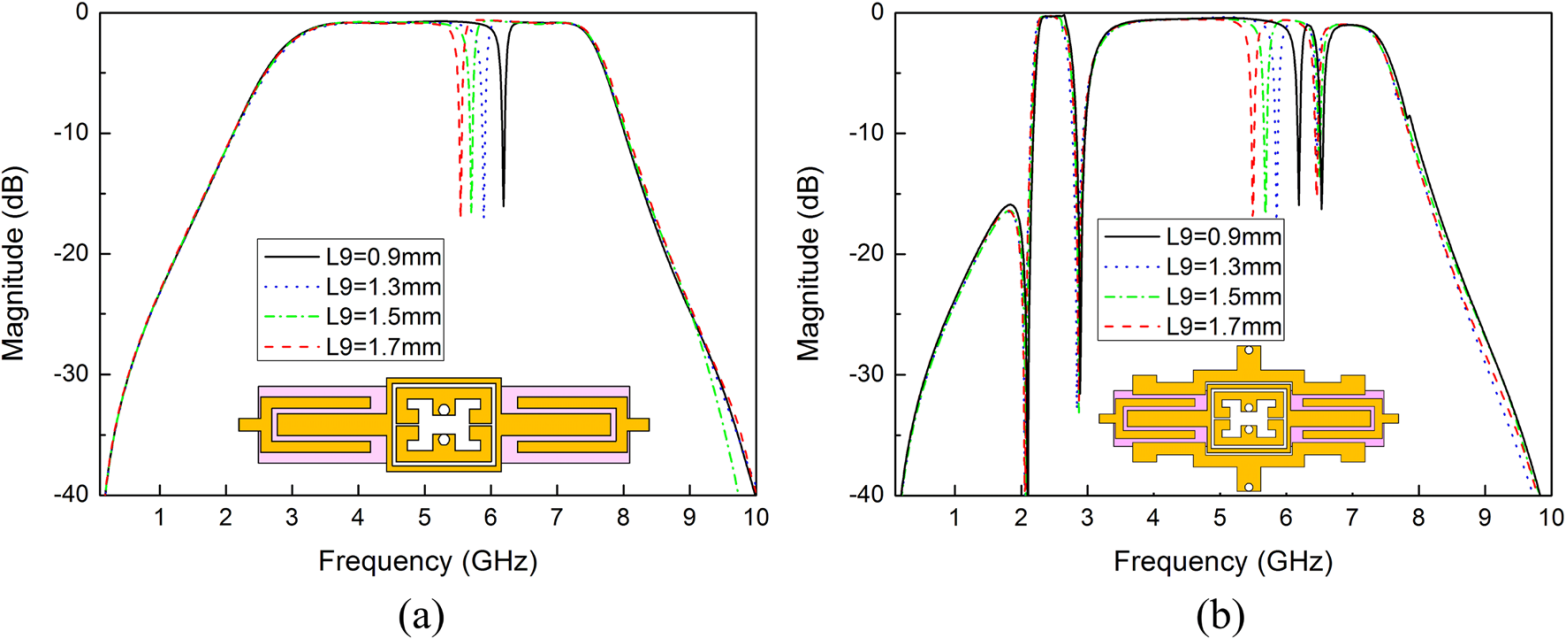
The variable second notch frequency corresponding to different length L9 (**a**) without and (**b**) with SSLSIR type2 loaded on the filter.

Similarly, the second notched band can be introduced by the shorted stub of SSLSIR type1 whose length is represented by L9 (about half wavelength with shorted end, combined with parasitic inductance of the via and mutual inductance between SLRR and SSLSIR type1). L9 can be used to adjust the second notched band as the length is relatively short, slight change has little effect on the resonant modes. As shown in Fig. 13a, the second notched band can be decreased by increasing the length L9, and the first and third notched band are not introduced because of SSLSIR type1 is embedded into the ring of SLRR without SSLSIR type2 loaded on the structure. This means that the three notched bands can appear independently in the wideband, depending on whether the two types of SSLSIRs are coupled with the SLRR. Fig. 13b reveals the transmission characteristic curve at different length L9 with both types of SSLSIRs configured in structure. It is easy to see that the three notched bands are formed in the wideband simultaneously, and the max attenuation of the center frequency of each notched band is better than − 15 dB, indicating that the performance meets the requirements for interference suppression.

**Figure 14:**
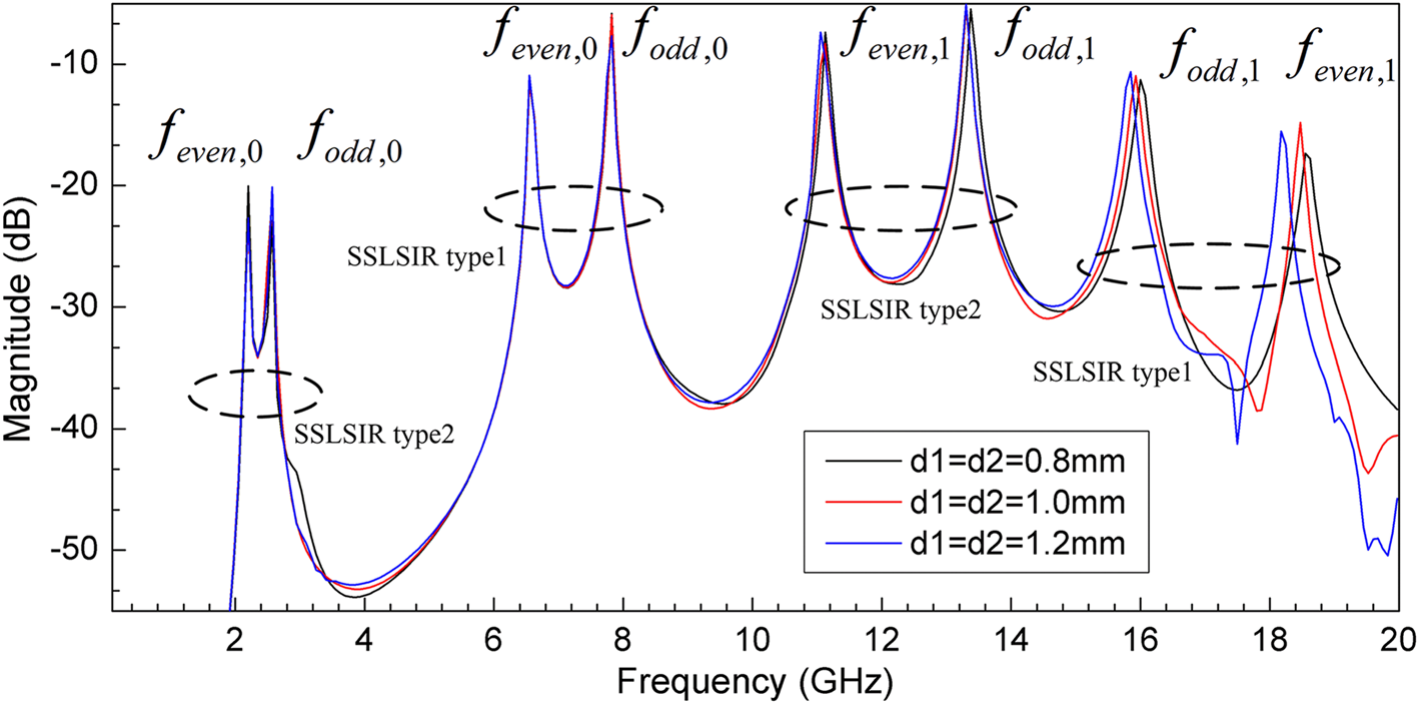
The mode distribution of the two types of SSLSIRs under different depth d1 and d2 of slit line.

SSLSIR not only provides resonant modes in the wideband, but also introduces additional spurious frequencies outside the band to increase the upper stopband bandwidth. Slit lines are loaded vertically on the low impedance section of each SSLSIR to enhance the upper stopband suppression. Figure 14 shows the effect of slit line on each resonant mode of the two types of SSLSIRs. The spurious modes ${\text{f}}_{{{\text{even,1}}}}$ ${\text{f}}_{{{\text{odd,1}}}}$ decrease with the increase of slit line depth d1 and d2 while the fundamental resonant modes ${\text{f}}_{{{\text{even,0}}}}$ and ${\text{f}}_{{{\text{odd,0}}}}$ remain almost unchanged, which verifies the characteristics of slit line discussed in the previous section.

**Figure 15:**
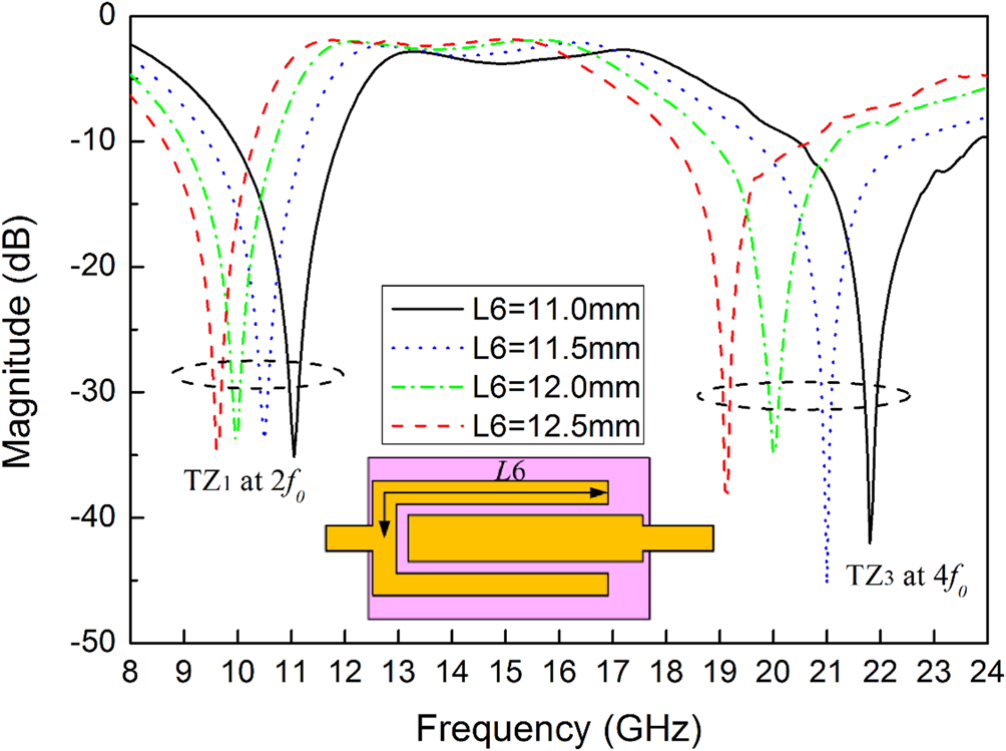
Variable TZ1 and TZ3 corresponding to different length L6.

There are four TZs outside the passband of the filter. The electrical length of the interdigital coupling line is set to ${{\lambda /4}}$ at the center frequency ${\text{f}}_{0}$, which will introduce TZ1 and TZ3 at ${\text{2f}}_{0}$ and ${\text{4f}}_{0}$, as shown in Fig. 15. The positions of TZ1 and TZ3 can be easily distributed by different length L6. TZ2 at 12.9 GHz and TZ4 at 30.6 GHz are generated by the shorted stubs of SSLSIR type2 and SSLSIR type1 represented by L3 and L9 whose electrical length is twice of the half-wavelength, respectively. Combined with the influence of slit line, the 1st even harmonics of SSLSIR type2 and 1st odd–even harmonics of SSLSIR type1 are adjusted to near TZ1 and TZ3, and the 1st odd harmonics of SSLSIR type2 and 1st parasitic band of SLRR are adjusted to TZ2 and TZ4, thus an enhanced upper stopband up to 32 GHz is achieved.

## Conclusion

This paper presents a miniaturized tri-notched wideband bandpass filter composed of SLRR and SSLSIR resonators. A wide passband ranging from 2.3 to 7.6 GHz is formed by coupling the open stub of SLRR with interdigital line. Two SSLSIRs with the same shape but different dimensions are placed inside and outside the ring of SLRR, and three controllable notched bands are introduced into the passband. Four TZs are located outside the passband of the filter, and slit lines are loaded vertically on the low impedance section of the SSSLSIR to enhance the upper stopband suppression with a − 20 dB upper stopband suppression up to 32 GHz. The proposed novel multi-functional filter can be applied to the situations where ultrawide stopband suppression, elimination of passband interference, miniaturization and wide passband are required at the same time.
